# Impact of Maternal Diet During Pregnancy on Allergic Predisposition in Offspring: Immune Programming Mechanisms

**DOI:** 10.34763/jmotherandchild.20252901.d-25-00016

**Published:** 2025-11-02

**Authors:** Maria Zofia Lisiecka

**Affiliations:** Department of Allergology, National Medical Institute of the Ministry of the Interior and Administration, Warsaw, Poland

**Keywords:** polyunsaturated fatty acids, cord blood, immune tolerance, pregnancy, probiotics

## Abstract

**Background:**

The aim of the study was to determine how dietary habits of pregnant women influence foetal immune programming and susceptibility to allergies in young children.

**Material and methods:**

The study followed 172 healthy women aged 20–38 years from early pregnancy to one year postpartum in Warsaw, Poland. Dietary data were collected at 12, 24, and 36 weeks using a validated questionnaire and three-day food diaries. Immunological analysis of newborn cord blood included multiplex cytokine analysis, flow cytometry, PCR, and pyrosequencing of FOXP3, IL-10, and TGFβ1 gene promoter regions.

**Results:**

The results showed an increased intake of polyunsaturated fatty acids, antioxidants and probiotics, accompanied by a decreased intake of allergenic foods. This was accompanied by a balanced immune profile in neonates: increased interferon gamma (IFN-γ) (12.36 pg/ml) and IL-10 (9.21 pg/ml), decreased interleukin 4 (IL-4) (5.82 pg/ml) and immunoglobulin E (IgE) (1.62 IU/ml), indicating a decrease in T helper 2 (Th2)-direction. Higher intake of omega-3 polyunsaturated fatty acids was linked to decreased FOXP3 methylation (r = −0.34; p = 0.008), and probiotics to IL-10 demethylation (r = −0.27; p = 0.03). By 12 months, 21.5% of children showed signs of allergic susceptibility, but those born to women with high omega-3 intake (> 13.5 g/day, OR = 0.42; p = 0.005) and probiotics (OR = 0.55; p = 0.027) had lower rates.

**Conclusion:**

The influence of maternal nutrition during pregnancy on the immune health of the child, in particular on the development of allergic predisposition, has been determined through the mechanisms of gene methylation and changes in the immune profile of newborns.

## Introduction

The rapid increase in the frequency of allergic diseases in early childhood is driving a growing scientific interest in prenatal factors capable of influencing the development of the foetal immune system. Particular attention is being paid to the diet of the pregnant woman as a key modifiable factor. It has been established that a deficiency of certain nutrients in the mother’s diet can disrupt the establishment of immunological tolerance in the foetus, contributing to the development of immune dysfunctions later in life. The early stages of embryogenesis and intrauterine development, when the mechanisms of immune regulation are laid down and the immune response is programmed, are most vulnerable to external influences, including dietary factors. Nutrients during this period can exert a long-term epigenetic influence on the expression of genes associated with immunity. However, despite the increasing number of studies, the precise role of individual dietary components in preventing allergic diseases in offspring remains insufficiently understood [1].

Given the high sensitivity of the foetal immune system to maternal factors, a deficiency of polyunsaturated fatty acids (PUFAs), particularly omega-3 PUFAs, can be considered a risk factor for disruptions in the development of immune defence. Jerzyńska et al. [2] conducted a study aimed at investigating the effect of omega-3 fatty acids in the diet of pregnant women on the development of the foetal immune system. The results showed that a low level of consumption of these substances correlated with increased expression of interleukin 4 (IL-4) and immunoglobulin E (IgE) in newborns, indicating a predominance of an allergic profile. However, the study did not take into account the possible influence of accompanying nutrients, such as antioxidants and prebiotics, which limits the generalisability of the findings.

Antioxidants play an important role in immune programming by protecting the developing foetal immune system from the damaging effects of oxidative stress [3]. It is known that pregnancy, especially in the third trimester, is accompanied by a physiological increase in the production of reactive oxygen species, which increases the need for antioxidant protection. A study by Michońska et al. [4] found that women with a deficiency of vitamins C and E in their diet had elevated levels of pro-inflammatory cytokines in their newborns. However, it should be noted that the sample included only pregnant women with chronic diseases, which may limit the generalisability of the data. Furthermore, this study did not consider the possible epigenetic pathways through which antioxidants can modulate the expression of genes involved in immune regulation. Alongside nutrients, researchers’ attention is drawn to the microbiota transmitted from mother to child. It has been established that probiotics consumed during pregnancy can modify the gut microbiota and promote the development of immune tolerance. Gao et al. [5] demonstrated that taking probiotics in the second and third trimesters was associated with a reduced frequency of eczema in children during the first year of life. However, the authors did not assess cytokine levels in umbilical cord blood and did not analyse the methylation of genes responsible for immune regulation, which leaves the question of the mechanisms of action open.

Vitamin D is considered a key modulator of innate and adaptive immune responses and plays an important role in the process of immune programming [6, 7]. Its deficiency during pregnancy is associated with an increased risk of developing asthma in children. A randomised controlled trial by Di Costanzo et al. [8] evaluated the effect of high-dose vitamin D supplementation on the frequency of allergic manifestations in offspring. The data obtained indicate a reduction in the frequency of asthma symptoms; however, the lack of analysis of potential interactions between vitamin D and other nutrients, such as fatty acids and antioxidants, makes it difficult to interpret the results in the context of the complex influence of diet on immune regulation. Epigenetic mechanisms, including the methylation of promoter regions of genes regulating the activity of T-regulatory cells (Treg cells), play a special role in the formation of immune tolerance. Bai et al. [9] investigated the association between the diet of pregnant women and epigenetic modification of forkhead box P3 (FOXP3) in newborns. It was shown that certain dietary components, particularly polyphenols, correlated with a reduction in the methylation of this gene, promoting the activation of Treg cells. However, the study did not include longitudinal follow-up of the children, which did not allow for the assessment of actual clinical outcomes.

The issue of consuming allergenic foods during pregnancy remains a matter of debate. On the one hand, excluding such foods may reduce the antigenic load on the foetus; on the other hand, it may contribute to a decrease in tolerance. Venter et al. [10] found that women who completely excluded peanuts, dairy products, and eggs had a higher risk of developing food allergies in their children. However, the study did not take into account individual characteristics of the microbiota and genetic predisposition, which limits the possibility of extrapolating the results to the general population. No less important is the influence of dietary patterns, in particular the frequency of meals and the balance of macronutrients. Venter et al. [10] and Coppola et al. [11] showed that irregular eating habits and a predominance of carbohydrates in the diet of pregnant women correlated with an increase in interleukin 6 (IL-6) levels in newborns, indicating a predisposition to a pro-inflammatory type of immune response. Nevertheless, the studies did not include indicators of epigenetic changes, which makes it impossible to analyse the mechanisms of long-term immune programming.

Thus, despite the growing number of studies, there remains insufficient data on the complex influence of various components of a pregnant woman’s diet on foetal immune programming and the development of allergic predisposition in children. In this regard, the aim of the study was to identify the relationship between the characteristics of maternal nutrition during pregnancy and the immune mechanisms that predetermine the development of allergies in offspring. The objectives of the study included assessing the nutrient composition of the diet, analysing the immune profile of newborns, and examining epigenetic changes in key immunoregulatory genes.

## Methods

The study was conducted from January 2023 to June 2024 at the Institute of Mother and Child in Warsaw, Poland. The prospective cohort study included 172 pregnant women aged 20 to 38 years (mean age 29.4 ± 4.1 years), followed up from the first trimester of pregnancy to one year postpartum. Participants were selected based on voluntary informed consent, absence of chronic diseases, immunopathologies, smoking, and alcohol consumption. Cases of gestational diabetes, pre-eclampsia, third trimester infections, use of systemic immunomodulators, and severe maternal or paternal allergies were excluded. All manipulations and procedures were performed according to the principles of the Declaration of Helsinki [12] and Good Clinical Laboratory Practice (GCLP) guidelines [13].

Dietary information was collected at the 12th, 24th, and 36th weeks of gestation using a validated Food Frequency Questionnaire (FFQ) technique [14] adapted to national dietary habits. FFQ results were compared with three-day food diaries completed by the participants during each period to verify macro- and micronutrient intake. A three-day food diary is a method used in nutrition studies where participants record everything they eat and drink over three days, including the quantity, type of food, and timing of consumption. This provides a more accurate representation of typical dietary habits than a single day’s record, as it accounts for day-to-day variations. Food diaries can be structured, with provided forms to guide participants, or unstructured, where participants write down their own entries. The data are analysed to assess nutrient intake, with tools or software used to calculate the nutritional content. While this method provides a detailed snapshot of eating patterns, it relies on participants’ accuracy and consistency, and bias may occur if foods are forgotten or portion sizes are misreported.

Special attention was paid to the content of fatty acids, antioxidants, probiotics, and allergenic foods (milk, nuts, fish). After delivery, within the first 15 min, cord blood was collected in a volume of 10 ml into sterile tubes with ethylenediaminetetraacetic acid (EDTA). Blood was centrifuged at 1500 g for 15 min at +4°C, after which serum and cells were separated and stored at −80°C. The concentrations of cytokines IL-4, interleukin 5 (IL-5), interleukin 10 (IL-10), IFN-γ, tumour necrosis factor alpha (TNF-α), and total IgE were determined using the LEGENDplex™ Human Th Cytokine Panel Multiplex Kit (13-plex) (BioLegend, USA). T-cell subpopulations were characterised by flow cytometry on a FACSCanto II device (BD Biosciences, USA) using labelled antibodies to CD3, CD4, CD8, CD25, FOXP3, and CD127. Cell viability was assessed using 7-AAD dye. Additionally, methylation of promoter regions of FOXP3, IL-10, and transforming growth factor beta 1 (TGFβ1) genes was analysed by pyrosequencing after bisulfite modification of deoxyribonucleic acid (DNA) isolated from cord blood mononuclear cells. Molecular studies were performed on a PyroMark Q24 system (Qiagen, Germany). Gene expression was measured by quantitative real-time PCR on a QuantStudio 5 instrument (Applied Biosystems, USA) using the TaqMan Gene Expression Assays probe system.

The selection of cytokines and immunological markers–IL-4, IL-5, IL-10, IFN-γ, TNF-α, and IgE–was based on their critical roles in immune responses and their relevance to allergic diseases, providing valuable insights into immune programming and allergic predisposition in offspring. IL-4 is central to the differentiation of T-helper 2 (Th2) cells and the stimulation of IgE production, a key marker of allergic sensitization. IL-5 plays a significant role in eosinophil activation, crucial for Th2-driven allergic inflammation. IL-10, an anti-inflammatory cytokine, regulates immune tolerance by inhibiting pro-inflammatory cytokines, helping maintain immune balance and preventing autoimmune responses. IFN-γ, produced by Th1 cells, counters Th2 responses and is involved in promoting a balanced immune system and protecting against allergies. TNF-α, a potent pro-inflammatory cytokine, is linked to tissue inflammation and immune dysregulation observed in allergic conditions. IgE serves as a direct marker of allergic sensitization and is crucial for understanding the risk of developing allergic diseases. This combination of pro-inflammatory cytokines, anti-inflammatory markers, and IgE levels offers a comprehensive assessment of immune response and the balance between immune activation and regulation, which are essential for understanding how maternal nutrition influences offspring’s allergic risk.

Allergic susceptibility in children was assessed at 6 and 12 months of age using skin prick tests with allergens (house dust, mites, birch pollen, cow’s milk protein; Stallergenes Greer, France) and serum specific IgE levels determined by ImmunoCAP (Thermo Fisher Scientific, USA). Parents completed the standardised International Study of Asthma and Allergies in Childhood (ISAAC) questionnaire modified for infants [15]. Statistical processing of data was performed using SPSS Statistics version 27.0 (IBM, USA). The student’s t-test or Mann–Whitney test was used to compare quantitative variables between groups, depending on the nature of the data distribution. The relationship between dietary components and immune parameters was assessed using the Spearman correlation coefficient. The influence of individual nutrients on the risk of allergic susceptibility was analysed by logistic regression with calculation of the odds ratio (OR) and 95% confidence interval. Differences were considered statistically significant at a significance level of *p* < 0.05.

## Results

Summary data from the FFQ and verification food diaries showed a consistent trend towards an increase in total caloric intake and a relative stabilisation of macronutrient levels. Particularly significant was the increased intake of PUFAs, antioxidants (vitamins C and E), and probiotic foods (fermented dairy products). However, there was a decrease in the consumption of traditional allergenic foods such as nuts and cow’s milk in the third trimester, which may indicate individualised strategies to reduce the risk of allergy in the offspring. The findings are summarised in Table 1 and will form the basis for further correlation with the immune parameters of the newborns.

**Table 1.** Average daily intake of key nutrients by pregnant women at different gestational ages (M ***±*** SD).

**Nutrient/Period**	**12th week**	**24th week**	**36th week**
Calories (kcal)	1985 ± 312	2137 ± 330	2215 ± 358
Protein (g)	78.2 ± 11.4	80.9 ± 12.1	81.7 ± 13.5
Fats (g)	76.5 ± 10.2	80.3 ± 11.8	84.1 ± 12.9
PUFA (g)	10.4 ± 2.1	12.7 ± 2.4	14.2 ± 2.7
Vitamin C (mg)	81.3 ± 19.2	93.8 ± 21.6	97.6 ± 22.1
Vitamin E (mg)	10.1 ± 2.5	11.7 ± 2.8	12.3 ± 3.0
Probiotics (servings/day)	0.8 ± 0.4	1.1 ± 0.5	1.4 ± 0.6
Nuts (servings/week)	3.2 ± 1.5	2.5 ± 1.2	1.8 ± 1.1
Cow’s milk (ml/day)	218 ± 75	184 ± 68	160 ± 62

*Source: D*eveloped by the author.

Analysis of the dynamics of nutritional behaviour of pregnant participants at different gestational periods revealed statistically and clinically significant changes in the consumption of biologically active nutrients affecting the immune programming of the foetus. The most pronounced trend was an increase in the intake of PUFAs, especially the omega-3 fraction. Average daily PUFA intake increased from 10.4 g at week 12 to 14.2 g at week 36, an increase of 36.5%. This figure reflects the increasing intake of foods rich in docosahexaenoic (DHA) and eicosapentaenoic (EPA) acids, particularly fatty marine fish and vegetable oils. These fatty acids are critical for neuroimmune development, participating in the synthesis of anti-inflammatory lipid mediators, resolvins, and protectins, which regulate the balance of T helper 1/T helper 2 (Th1/Th2) cells and may reduce the risk of allergic sensitisation in offspring. In addition to fatty acids, pregnant women showed a sustained increase in antioxidant intake: vitamin C levels increased by 16.3 mg and vitamin E by 2.2 mg between the first and third trimesters. These nutrients have a marked ability to neutralise reactive oxygen species that can damage rapidly proliferating cells of the foetal immune system. Antioxidant deficiency during critical periods of embryogenesis may lead to adverse activation of NF-κB signalling pathways and increased production of pro-inflammatory cytokines, potentially contributing to an atopic phenotype [16, 17].

The significant increase in the consumption of probiotic foods such as yogurt and kefir, especially by the third trimester (up to 1.4 servings per day on average), reflects the important role of informed food choices. These foods have the potential to modulate the composition of the maternal gut microbiota, which in turn affects the metabolic profile of microbial metabolites, including short-chain fatty acids (SCFAs) such as butyrate. Butyrate, according to current data, enhances FOXP3 gene expression in regulatory T cells and promotes tolerance in the foetus.

The opposite trend was demonstrated by the consumption of allergenic foods. The number of servings of nuts decreased from 3.2 to 1.8 per week, and the volume of cow’s milk consumption decreased by 58 ml per day by the end of pregnancy. Although these behaviours may be driven by perceptions of allergy prevention, they remain controversial from an evidence-based perspective. Reducing exposure to allergens *in utero* potentially impairs the formation of oral and enteric tolerance [18]. In addition, exclusion of these foods may limit the intake of essential nutrients–amino acids, calcium, B and D vitamins–with implications for both immune and neurobehavioural regulation of the foetus. Thus, the nutritional profile of pregnant women demonstrates a complex and adaptive nature, reflecting both a desire to improve nutrient status and avoidance behaviour towards potential allergens. This dual strategy deserves detailed consideration in light of further immunological and epigenetic data.

Restricting the consumption of allergenic foods during pregnancy is generally considered an effective strategy for reducing the risk of allergies in children. The results of this study demonstrate a somewhat paradoxical effect. Avoiding allergens during intrauterine development may interfere with the formation of oral and enteral tolerance. Under normal conditions, when exposed to allergens during development, a baby’s body has a chance to develop tolerance, which is the basis for an adequate immune response in the future. The lack of this contact can lead to the child’s immune system not “recognizing” certain foods as harmless, which increases the likelihood of developing allergic reactions after birth. In addition, excluding these foods may limit the intake of nutrients important for the health of the mother and child, such as amino acids, calcium, and vitamins B and D, which are important not only for the normal functioning of the immune system, but also for the neurobehavioural development of the foetus. These factors may explain the complexity and adaptability of pregnant women’s dietary strategies and highlight the importance of further study of this issue from the perspective of immunology and epigenetics.

Immunological analysis of cord blood has allowed quantification of key mediators of the innate and adaptive immune response in neonates. Particular attention was paid to Th1- and Th2-type cytokines, regulatory molecules, and total IgE level as an indicator of propensity to atopic sensitisation. The data obtained reflect the initial state of the immune system at birth and serve as an important biomarker of intrauterine programming. Table 2 shows the average values of concentrations of cytokines IL-4, IL-5, IL-10, IFN-γ, TNF-α, and total IgE in cord blood serum obtained from newborns in the first minutes after birth.

**Table 2.** Concentrations of immune markers in cord blood of newborns (n=172, M ± SD).

**Indicator**	**Concentration (pg/ml) or IU/ml**
IL-4	5.82 ± 2.14
IL-5	3.47 ± 1.78
IL-10	9.21 ± 3.15
IFN-γ	12.36 ± 4.27

TNF-α	8.75 ± 2.63
Total IgE (IU/ml)	1.62 ± 0.89

*Source: D*eveloped by the author.

Immunological mapping of newborns according to cytokine analysis of umbilical cord blood serum revealed characteristic patterns of innate and adaptive immune activity at the time of birth, reflecting the intrauterine conditions of immune system programming. The most pronounced result was the relative predominance of pro-inflammatory Th1-type cytokines–IFN-γ and TNF-α–over classical Th2 mediators, including interleukin-4 (IL-4) and interleukin-5 (IL-5). The mean concentration of IFN-γ (12.36 pg/ml) was almost double that of IL-4 (5.82 pg/ml), which may indicate a predominance of the cell-mediated immune pathway and a more balanced immune setup at the time of birth. This profile is characteristic of neonates with intrauterine exposure to factors that promote maturation of the innate immune response, including microbial components, SCFA products of probiotic metabolism and PUFAs consumed by the mother. IFN-γ stimulates the differentiation of CD4+ T cells towards a Th1 phenotype and also activates NK cells, enhancing antiviral and antitumour defence. Moderate levels of TNF-α (8.75 pg/ml) complemented this picture by supporting the proliferation of macrophages and dendritic cells involved in antigen presentation and immune memory formation. TNF-α levels, being within physiological limits, did not reach values greater than 11.7 pg/ml, which is associated with systemic inflammation or intrauterine infection.

The high concentration of IL-10 (9.21 pg/ml) is of particular interest in terms of regulation of immune balance. IL-10, secreted by Treg cells and other regulatory populations, inhibits the expression of pro-inflammatory cytokines and costimulatory molecules, providing immune tolerance to autoantigens and a safe response to food antigens. Its stable levels in the cohort may indicate an active induction of immunoregulation in response to metabolic signals, including butyrate and acetate produced by fermentation of probiotic substrates in the maternal gut. In contrast, relatively low levels of IL-4 and IL-5 support the hypothesis of delayed activation of the Th2 pathway in the absence of intrauterine sensitisation to allergens. IL-4 played a key role in the induction of isotypic switch to IgE synthesis in B cells, and IL-5 regulates eosinophil activation and survival. Suppression of these markers may be due to both maternal nutrition (restriction of allergenic foods, intake of PUFAs and probiotics) and genetic mechanisms, including epigenetic regulation of promoter regions of Th2-cytokine genes.

Total IgE levels were 1.62 IU/ml on average, which is within the normal range for non-atopic neonates. However, approximately 19% of the children had values exceeding 2.5 IU/ml, which is considered a potential marker of increased risk of allergy according to Jerzyńska et al. [2]. These cases will be discussed in more detail in Section 4, in the context of prick test data and specific IgE. It should be noted that high IgE levels at birth are often associated with maternal factors, including antioxidant deficiency, high intake of sugars and fats, and intrauterine inflammation. Overall, the cord blood immune profile reflects a complex balance between protective activation of the Th1 pathway and maintenance of regulatory tolerance [19]. It showed high individual variation and provides a basis for further analysis, in combination with epigenetic and clinical data.

Methylation is a process in which a methyl group (–CH_3_) is attached to a DNA molecule, changing gene activity without changing its sequence. Methylation usually suppresses gene activity, preventing it from being actively expressed. This is an important mechanism of epigenetic regulation that controls which genes will be active and which will not. In the context of allergies, methylation plays a role in regulating the immune response, particularly in the function of Tregs, which help prevent excessive immune reactions to harmless substances such as pollen or food. If methylation in genes that control Treg function (e.g., FOXP3, IL-10, TGFβ1) increases, it can reduce gene activity, contributing to the development of allergies. Therefore, changes in gene methylation may be an important factor in the development of allergic diseases, and these changes may be caused by factors such as the mother’s diet during pregnancy.

Epigenetic analysis of methylation of promoter regions of key immunoregulatory genes in cord blood mononuclei made it possible to assess the influence of nutrient environment on the mechanisms of transcriptional regulation. The genes FOXP3, IL-10, and TGFβ1, which play a central role in the induction and maintenance of immune tolerance, especially through Treg cell activation, were selected. Methylation levels of promoter sites were determined by pyrosequencing after bisulfite modification of DNA and are presented in Table 3.

**Table 3.** Mean methylation levels of promoter regions of immune genes in cord blood mononuclear cells (n = 172, M ± SD) and their correlation with nutrients.

**Gene**	**Methylation Level (%) (M ± SD)**	**Correlation with PUFAs (r/p)**	**Correlation with Probiotics (r/p)**	**Correlation with Antioxidants (r/p)**
FOXP3	18.4 ± 5.2	**–0.34/0.008**	–0.12/0.146	–0.18/0.062
IL-10	12.7 ± 4.6	**–0.09/0.193**	–0.27/0.030	–0.15/0.081
TGFβ1	9.3 ± 3.1	**–0.14/0.097**	–0.10/0.176	–0.26/0.018

*Source: D*eveloped by the author.

The results of the analysis of methylation levels of promoter regions of FOXP3, IL-10, and TGFβ1 genes in cord blood mononuclear of newborns demonstrate a sensitive relationship between the nutrient profile of maternal nutrition and epigenetic tuning of regulatory links of the foetal immune system. These genes are central components in the formation of immune tolerance, activation of Treg cells and suppression of excessive production of pro-inflammatory cytokines, which is critical for the prevention of atopic and autoimmune conditions. The FOXP3 gene, encoding a key transcriptional regulator of Treg cells, had an average methylation level of 18.4 ± 5.2%. However, when stratified by individual values, significant variations were found: 22% of newborns had methylation levels exceeding 22%, which may be associated with transcriptional suppression and reduced functionality of Treg populations. When analysing associations with maternal diet, we found that women whose omega-3 PUFA intake exceeded 13 g/day in the third trimester had significantly lower levels of FOXP3 promoter methylation (by 3.1%, *p* < 0.01) in their offspring. This confirms the role of omega-3 fatty acids as epigenetic modifiers that promote chromatin opening and maintain the transcriptionally active state of immune tolerance regulation genes. Biochemically, this effect may be realised through the interaction of PUFAs with nuclear receptors, including peroxisome proliferator-activated receptor gamma (PPARγ) and retinoid X receptors (RXRs), which in turn affect the activity of DNA methyltransferases and histone deacetylases.

A mean methylation level of 12.7 ± 4.6% was found for IL-10, consistent with moderate transcriptional activity. However, when the groups were compared in terms of probiotic intake (over 1 serving/day in the 2nd and 3rd trimesters), a significant difference was recorded: IL-10 methylation was 2.8% lower in the probiotic group (*p* = 0.03). This suggests the participation of microbial metabolites, primarily butyrate and propionate, in the regulation of IL-10 expression through demethylating action and activation of G-protein coupled receptors (GPRs), specifically, GPR43 and GPR109A signalling pathways. Given that IL-10 played a key role in limiting the Th2 response and inducing B-cell anergy, reducing its epigenetic suppression may represent an important factor in preventing immune system hyperresponsiveness in offspring.

The promoter region of TGFβ1 showed the lowest level of methylation (9.3 ± 3.1%), which correlated with a high basal level of transcription of this cytokine. When stratified by the level of antioxidants in the maternal diet, a moderately significant negative correlation was found between the intake of vitamins C and E and the degree of TGFβ1 methylation (Spearman r = −0.26; *p* = 0.018). This suggests that antioxidants may be involved in protecting promoter sites from oxidative damage and thereby contribute to maintaining the active transcriptional status of the gene. TGFβ1 is critical for stabilising the Treg phenotype and suppressing the Th17 response involved in the pathogenesis of inflammatory diseases. Cumulatively, the present data emphasise that even moderate variations in the composition of a pregnant woman’s diet can induce significant epigenetic effects in foetal immune cells. This confirms the concept of “nutriepigenetic programming” and opens prospects for the introduction of personalised nutritional strategies aimed at the prevention of immune disorders already at the intrauterine stage of development.

The results of clinical and immunological monitoring of children aged 6 and 12 months demonstrated an increasing tendency to develop sensitisation and clinical manifestations of allergy during the first year of life. Sensitisation was determined on the basis of positive skin prick tests and/or elevated levels of specific IgE (≥ 0.35 kA/L) to household and food allergens. Parental complaints recorded using the modified ISAAC questionnaire, including skin symptoms, wheezing and rhinorrhoea, were also considered. The cumulative data are presented in Table 4 and reflect both immunological and clinical features of allergic susceptibility in infants.

**Table 4.** Frequency of sensitisation and clinical manifestations of allergy in children at 6 and 12 months of age (n = 172).

**Indicator**	**6 months (%)**	**12 months (%)**
Positive prick test ≥ 1 allergen	11.0	17.4
Elevated specific IgE (≥ 0.35 kA/L)	9.9	16.3
Sensitisation to cow’s milk protein	6.4	9.3
Sensitisation to dust mites	3.5	7.0
Sensitisation to birch pollen	1.7	4.1
Sensitisation to house dust	4.7	8.1
Atopic dermatitis (according to ISAAC)	7.6	11.6
Respiratory symptoms of allergy (wheezing, rhinitis)	5.2	9.8
General allergic predisposition*	13.4	21.5

*Note:*

* Predisposition was defined when at least one of three criteria was present: positive prick test, elevated IgE, clinical symptoms.

*Source:* Developed by the author.

The results of allergic status assessment in infants aged 6 and 12 months made it possible to trace the early dynamics of formation of an immune phenotype predisposed to the development of allergy. The frequency of sensitisation, confirmed by both prick tests and levels of specific IgE, increased with age, reaching 17.4% and 16.3%, respectively, by 12 months of age. Among allergens, cow’s milk protein remained the most clinically important, with sensitisation to it observed in 9.3% of children. In some cases, multiple sensitisations were registered in infants, mainly to food and household allergens. In parallel, the frequency of clinical manifestations increased: symptoms of atopic dermatitis and respiratory allergy were observed in 11.6% and 9.8% of children, respectively. These data confirm that the early postnatal period is a critical window for the realisation of immune programming initiated *in utero*.

Other potential factors that may influence the development of allergies in children include environmental influences, particularly allergens such as pollen, house dust, air pollution, and other environmental factors. Sensitization to birch pollen and house dust, detected through skin prick tests, indicates that allergies may be caused not only by genetic or dietary factors, but also by the presence of allergens in the environment. Exposure to these allergens at an early age can activate a child’s immune system, increasing the risk of developing allergic reactions such as rhinitis, bronchial asthma, and atopic dermatitis. Other environmental factors, such as air pollution, can also modulate the immune response and contribute to the development of allergies by affecting the body’s ability to control inflammation and regulate allergic reactions [20,21,22].

An increased intake of omega-3 PUFAs by the mother, especially in the third trimester of pregnancy, demonstrated a significant role in reducing the risk of allergy in the offspring. According to regression analysis, women with a high PUFA intake (more than 13.5 g per day) were significantly less likely to develop sensitisation in their children (OR = 0.42; 95% CI, 0.22–0.78; *p* = 0.005). The putative mechanism of action includes the ability of omega-3 PUFAs to inhibit Th2 cell activation and enhance the expression of immunoregulatory genes such as FOXP3 and IL-10 by reducing their methylation levels. In addition, these fatty acids are involved in the synthesis of resolvins and protectins, lipid mediators that contribute to the reduction of IgE production and inhibition of mast cell degranulation.

Regular consumption of probiotics also had a positive effect. Children whose mothers consumed fermented dairy products ≥ 1 serving/day in the second and third trimesters were less likely to have manifestations of atopic dermatitis and respiratory allergy (OR = 0.55; 95% CI, 0.31–0.94; *p* = 0.027). The likely mechanism for this effect involves modulation of the maternal microbiota with subsequent foetal exposure via SCFAs, especially butyrate, which has proven potential to activate Treg cells and reduce epigenetic suppression of IL-10. These children also exhibited reduced levels of total IgE and an increased IL-10/IL-4 ratio in cord blood, reflecting a mounting regulatory rather than allergic immune response.

The opposite effect was reported in subgroups of mothers who practised avoidance behaviour towards allergenic foods. Specifically, nut consumption of less than one serving per week during the second and third trimesters of pregnancy was significantly associated with an increased risk of food sensitisation in the child (OR = 1.76; 95% CI, 1.01–3.05; *p* = 0.042), indicating a statistically significant association. This relationship persisted after adjustment for potential confounders (maternal age, gestational age, prepregnancy BMI, education level, and presence of pets). Thus, limiting exposure to food antigens during critical periods of antigen presentation may impair the process of immune tolerance formation and lead to a shift towards a Th2 response. A similar, also statistically significant trend was observed when cow’s milk intake was restricted (< 150 ml/day): in children from this subgroup, sensitisation to cow’s milk protein was more frequent (OR = 1.93; 95% CI, 1.04–3.62; *p* = 0.038), and total IgE levels were higher by 0.48 IU/ml on average (*p* = 0.021). This may be a consequence of the deficiency of immunomodulatory components of dairy products (lactoperoxidase, immunoglobulins, casein), as well as attenuation of microbial stimulation, as evidenced by reduced IL-10 production and a tendency to hyperreactivity of the immune response.

The findings support the concept of nutrient-dependent immune programming, in which maternal diet during pregnancy has a lasting effect on the child’s immune profile. PUFAs and probiotics are the most protective factors, while excessive allergen avoidance may, in contrast, increase the risk of sensitisation. These results form the basis for the development of personalised nutritional strategies for antenatal allergy prevention. Healthcare professionals can provide pregnant women with recommendations on dietary restrictions that will help strengthen immune health and reduce the risk of allergies in children. It is important to avoid unnecessary restrictions on the consumption of potentially allergenic foods such as milk, nuts, and fish, as this may interfere with the development of tolerance in the child. Instead, women should follow a balanced diet that includes these foods, unless the mother has a known allergy. It is important to include omega-3 fatty acids, particularly DHA and EPA, in the diet, as these substances support the development of the child’s immune system and may reduce the risk of allergic diseases. It is also recommended to consume foods rich in antioxidants, such as vitamins C and E, which help reduce oxidative stress and support the health of the immune system.

Probiotic foods, such as yogurt and kefir, can help balance the mother’s intestinal microflora, which in turn has a positive effect on the development of the child’s immune system. Pregnant women should also ensure adequate intake of vitamins and minerals, particularly vitamins B, D, calcium, and magnesium, which are essential for the normal development of the foetal immune system. Finally, it is important to avoid unnecessary dietary restrictions without medical indications, as this can lead to nutrient deficiencies that negatively affect the health of both the mother and the child.

## Discussion

This research comprehensively characterises the influence of changes in pregnant women’s dietary behaviour on foetal immune programming. Of particular significance is the identified positive trend in the consumption of PUFAs, antioxidants, and probiotic products. These nutrients, as confirmed by several independent studies, may exert a modulating effect on the developing immune system, reducing the risk of allergic diseases in the postnatal period.

The noted increase in omega-3 PUFA consumption, demonstrated in the third trimester, aligns with the findings of Kim [23], who showed that the intake of more than 13 g/day of DHA and EPA in late gestation is associated with increased resolvin expression and reduced IgE production. Similar conclusions are drawn by Gupta et al. [24], highlighting the role of omega-3 fatty acids in enhancing regulatory T-cell differentiation. However, Bai et al. [25] note the absence of a significant reduction in the incidence of atopic dermatitis with PUFA consumption, which may be due to differences in the study population and methodology. The present research distinguishes itself by employing direct epigenetic and immunological markers, thereby providing a more objective interpretation of the results.

The increased consumption of antioxidants, particularly vitamin C and vitamin E, reflects an important trend towards heightened nutritional awareness among pregnant women [26,27,28]. According to data from Ogawa et al. [29], adequate levels of these vitamins prevent the activation of signalling pathways associated with NF-κB, reducing the production of pro-inflammatory cytokines. Lee et al. [30] emphasise that vitamin E can enhance IL-10 synthesis, promoting the development of immune tolerance. At the same time, Venter et al. [31] point to the limited bioavailability of these vitamins under conditions of high oxidative stress. Nevertheless, the presented study demonstrates a consistent decrease in TGFβ1 promoter methylation in newborns with high antioxidant intake, confirming their involvement in the epigenetic regulation of the immune response.

The consumption of probiotic products showed a positive correlation with the epigenetic activation of the IL-10 gene, which coincides with the findings of Grijincu et al. [32], who described the mechanism of action of butyrate on the expression of immunoregulatory cytokines via GPR receptors. These receptors, particularly GPR43 and GPR109A, are activated by SCFAs and trigger signalling cascades that promote the transcriptional activation of IL-10 and FOXP3. Similar results have been reported by Jiao et al. [33], who showed a reduction in the frequency of allergic manifestations in infants whose mothers regularly consumed fermented dairy products. These children exhibited not only lower levels of IgE but also an increased IL-10/IL-4 ratio, indicating a predominance of a regulatory profile. Idris et al. [34], in contrast, argue that the clinical effect of probiotics is not reproducible under conditions of a diverse diet, where the influence of probiotic products may be negated by other dietary factors. However, the present study maintained clear stratification by the level of probiotic consumption, with differentiation by frequency and type of products (kefir, yogurt, acidophilus drinks), allowing for a more accurate assessment of this nutrient’s contribution to immune programming. Additionally, the identified link between probiotic consumption and reduced IL-10 promoter methylation underscores not only the associative but also the potentially causal nature of the observed changes. This provides a basis for considering probiotic products not simply as a dietary component but as an active tool for immunomodulation with an epigenetic effect, particularly significant in the context of early foetal development.

The reduced consumption of traditionally allergenic foods, such as nuts and cow’s milk, observed in the third trimester, suggests the presence of conscious behavioural strategies. According to Nelson and Friedman [35], avoidant dietary behaviour may reduce the antigenic load on the foetus and thereby prevent sensitisation. However, Coronado [36] and Zainal et al. [37] point to the opposite effect: limiting contact with food antigens *in utero* may disrupt the development of oral and enteral tolerance. The data from the present study confirm this hypothesis: children of women with minimal consumption of nuts and milk had higher levels of total IgE and an increased frequency of sensitisation. The immune profile of newborns, obtained through the analysis of cytokines and total IgE, demonstrates a predominance of the Th1 response, which aligns with the concept of early maturation of innate immunity. Xie et al. [38] showed that high levels of IFN-γ in umbilical cord blood correlate with a reduced risk of respiratory allergy. Zhao et al. [39] also highlight the role of TNF-α in the formation of antigen-presenting activity and the development of immune memory. Conversely, Reiter et al. [40] suggest that excessive activation of the Th1 pathway may increase the risk of autoimmune diseases. However, in this study, cytokine levels were within the physiological range, indicating a balanced immune response.

High levels of IL-10 with a simultaneous decrease in IL-4 and IL-5 in newborns suggest the activation of regulatory mechanisms aimed at maintaining immune balance and limiting hyperreactive responses [41, 42]. Such a cytokine profile may indicate the dominance of Treg-mediated immune regulation at birth. Balla et al. [43] described a similar profile in children born to mothers with diets enriched in SCFAs and omega-3 PUFAs, emphasising the influence of microbial metabolites on IL-10 expression and the suppression of the Th2 response. Similar results have been presented by Lim et al. [44], who note the importance of IL-10 for the formation of B-cell anergy and the prevention of excessive IgE production. At the same time, Damron et al. [45] criticise the use of IL-10 as the sole marker of tolerance, considering its level dependent on nonimmune factors, including the mother’s metabolic state and perinatal stressors. Nevertheless, the correlation between reduced IL-10 methylation and high probiotic consumption in the present study strengthens the arguments in favour of the immunoregulatory effect of maternal diet, particularly in the aspect of epigenetic programming, confirming the functional significance of IL-10 as a component of a stable regulatory response. Furthermore, it has been established that IL-10 can exert a modulating influence on the development of the gut microbiota in newborns, promoting colonisation by probiotic strains and reducing the risk of dysbiosis in the early postnatal period.

The analysis of FOXP3 gene methylation showed significant sensitivity to the consumption of omega-3 fatty acids, which confirms the data of Wu et al. [46], highlighting the role of PUFAs as epigenetic modulators. Hanson et al. [47] also emphasise the involvement of PPARγ receptors in the regulation of FOXP3 activity. However, Han et al. [48] did not find significant changes in FOXP3 methylation levels in response to dietary interventions, which may be related to differences in the timing of the analysis. In contrast, the present study was conducted during the critical period of the end of pregnancy, when the epigenetic stability of the Treg phenotype is established.

Finally, clinical observation data at 6 and 12 months confirmed that the mother’s nutrient profile has a long-lasting effect on the child’s allergic predisposition. The increasing number of cases of sensitisation and clinical manifestations of allergy in infants, especially to cow’s milk protein and household allergens, was found to be statistically significantly associated with the level of consumption of key nutrients during pregnancy. This suggests the presence of a delayed effect of nutrient exposures, persisting throughout the first year of life. These results confirm the thesis of Pretorius et al. [49] that the prenatal diet represents a critical point of intervention for reducing the risk of immune disorders and underscore the importance of early detection and targeted intervention for nutrient deficiencies or excessive avoidance. The data obtained may form the basis for the development of personalised nutritional strategies during the gestational period, aimed at reducing immune hyperreactivity, establishing stable tolerance, and preventing allergies, as well as maintaining the functional maturity of the immune system in children.

In conclusion, the comprehensive analysis of pregnant women’s dietary behaviour and its impact on the offspring’s immune system has revealed a number of significant associations, confirming the concept of nutrient-dependent programming. The most favourable factors were the consumption of omega-3 PUFAs and probiotics, providing epigenetic activation of regulatory genes and reducing sensitisation in children. Excessive avoidance of allergens, on the other hand, may disrupt the development of immune tolerance. The results obtained underscore the need to develop personalised nutritional strategies during pregnancy and indicate the promise of further research, including genetic, microbiome, and metabolomic parameters of the mother and child.

## Conclusions

This study made it possible to comprehensively characterise changes in the nutritional behaviour of pregnant women at different gestational periods and their impact on the immune profile of newborns. It was found that during pregnancy there is a gradual increase in daily caloric intake accompanied by an increase in the intake of key nutrients such as PUFAs, vitamins C and E, and probiotic products. The increase in omega-3 PUFA intake was particularly pronounced, accompanied by a decrease in the degree of FOXP3 gene methylation in cord blood mononuclear cells and may indicate the formation of a more pronounced immunoregulatory potential in newborns.

Immunological mapping showed a predominance of Th1-type mediators, particularly IFN-γ and TNF-α, as well as moderately high levels of the anti-inflammatory cytokine IL-10. Low concentrations of IL-4 and IL-5 and physiological levels of total IgE in the majority of newborns suggest the absence of intrauterine sensitisation and the presence of a balanced setting of the immune system towards anti-infective and regulatory activity. These data confirm the protective significance of the nutrient background of pregnancy in the formation of innate and adaptive immune response. Analysis of methylation of promoter regions of FOXP3, IL-10, and TGFβ1 genes revealed significant correlations with nutritional components. Higher PUFA intake was associated with reduced FOXP3 methylation, regular consumption of probiotics was associated with increased transcriptional availability of IL-10, and antioxidants showed an association with the active status of the TGFβ1 gene. These results suggest the existence of a mechanism of nutriepigenetic programming of foetal immune tolerance.

Monitoring of children at 6 and 12 months of age confirmed that nutrient saturation of the maternal diet with PUFAs and probiotics was associated with a lower incidence of allergic sensitisation and clinical manifestations of atopy. At the same time, limitation of potential food allergens in the maternal diet, on the contrary, was accompanied by an increase in the risk of sensitisation in children, which suggests a possible loss of induction of immune tolerance with insufficient antigenic exposure during the intrauterine period. Thus, the obtained data confirm the concept of nutrient-dependent programming of the foetal immune system and emphasise the importance of a balanced diet of a pregnant woman as a factor in reducing the risk of allergy in the child. The development of personalised nutritional strategies that include enrichment of the diet with omega-3 fatty acids and probiotics while maintaining moderate exposure to food antigens is a promising direction.

A limitation of the present study is the lack of consideration of genetic predisposition factors for allergy, and limited information on exposure to postnatal environmental factors. Longitudinal studies with an expanded sample and inclusion of epigenetic, clinical, and microbiome parameters later in development are needed to improve the validity of the findings.

### Key points

1. The study explored how maternal diet impacts fetal immune programming and allergy risk in children.
2. A diet high in polyunsaturated fatty acids and probiotics reduced allergy markers in newborns.
3. Omega-3 intake and probiotics were linked to epigenetic changes in immune-regulatory genes.
4. Higher intake of these nutrients led to lower allergy susceptibility in children by 12 months.
